# Fast Determination of Yttrium and Rare Earth Elements in Seawater by Inductively Coupled Plasma-Mass Spectrometry after Online Flow Injection Pretreatment

**DOI:** 10.3390/molecules23020489

**Published:** 2018-02-23

**Authors:** Zuhao Zhu, Airong Zheng

**Affiliations:** College of Ocean and Earth Sciences, Xiamen University, Xiamen 361102, China; zhz@stu.xmu.edu.cn

**Keywords:** rare earth elements, flow injection, inductively coupled plasma-mass spectrometry, seawater

## Abstract

A method for daily monitoring of yttrium and rare earth elements (YREEs) in seawater using a cheap flow injection system online coupled to inductively coupled plasma-mass spectrometry is reported. Toyopearl AF Chelate 650M^®^ resin permits separation and concentration of YREEs using a simple external calibration. A running cycle consumed 6 mL sample and took 5.3 min, providing a throughput of 11 samples per hour. Linear ranges were up to 200 ng kg^−1^ except Tm (100 ng kg^−1^). The precision of the method was <6% (RSDs, *n* = 5), and recoveries ranged from 93% to 106%. Limits of detection (LODs) were in the range 0.002 ng kg^−1^ (Tm) to 0.078 ng kg^−1^ (Ce). Good agreement between YREEs concentrations in CASS-4 and SLEW-3 obtained in this work and results from other studies was observed. The proposed method was applied to the determination of YREEs in seawater from the Jiulong River Estuary and the Taiwan Strait.

## 1. Introduction

Due to their similar chemical properties, yttrium and fourteen rare earth elements (La, Ce, Pr, Nd, Sm, Eu, Gd, Tb, Dy, Ho, Er, Tm, Yb, Lu), collectively named YREEs, have always been studied together. Rare earth elements have a narrow range of relative atomic weights, ranging from 138.91 (La) to 173.04 (Lu), which results in extremely coherent chemical properties [1,2]. YREEs in seawater are drawing increasing attention due to the following aspects: (1) YREEs can be used as powerful tracers in marine biogeochemistry (ocean circulation, scavenging processes, trace metal cycles, etc.) and the redox-sensitive nature of Ce and Eu make them valuable for oxidation-reduction reactions [1,2,3,4,5,6]; (2) as YREEs are analogues of the radioactive actinides, understanding the geochemical cycling of YREEs can provide clues on the behavior of actinides (Am and Cm), which is important for monitoring the migration of actinides in radioactive waste repositories [7,8]; and (3) YREEs are widely used in industry (superconductor, functional materials, etc.), medical diagnostics (MRI, magnetic resonance imaging) and agriculture (as fertilizer) [9,10,11,12,13,14]. Anomalous concentrations of Gd, La and Sm discovered in estuarine and coastal seawaters reveals that the risks of YREEs’ release into the ocean through runoff and sewage are increasing, threatening marine ecosystems and altering the distribution patterns of YREEs in estuaries and oceans [2,9,10,11,12,13,14]. Therefore, it is of great interest to monitor YREEs in seawater. 

However, quickly and accurately determining the concentration of YREEs in seawater is a challenge because of their low concentrations and high salt matrix. The emergence of inductively coupled plasma-mass spectrometry (ICP-MS) has made trace and multi-element analysis more readily available and has been commonly used for the determination of YREEs. Nevertheless, little tolerance to total dissolved solids (<0.1%) makes direct (or after dilution) determinations of YREEs in seawater by ICP-MS inadvisable, since large amounts of salts will not only cause clogging of nebulizer, torch and cones, leading to signal drift, but also introduce severe polyatomic interferences. Thus YREEs must be separated and concentrated before detection. As pretreatment procedures, liquid-liquid extraction [5,15,16,17], co-precipitation [18,19,20,21], and solid-phase extraction [22,23,24,25,26,27] techniques have been utilized. Among these procedures, solid-phase extraction using chelating absorbents is becoming popular due to its wide selectivity of YREEs, low risk of contamination, freedom from toxic reagents and its simplicity for interfacing with ICP-MS to permit online determination when using flow injection (FI) as sample preconcentration systems. Compared to offline determination (batch method), online approaches using FI-ICP-MS has the advantages of sensitive response, high sample throughput, small volumes consumption of sample and reagents, little risk of contamination and labor savings [9,12,28,29,30,31,32,33,34,35,36]. The commercially available FIAS-400 (Perkin-Elmer, Waltham, MA, USA) [29,31,32,33] and SeaFAST (Elemental Scientific, Omaha, NE, USA) [36] FI system has been used in many studies devoted to the development of online determination of YREEs in seawater when coupled with ICP-MS. However, the relative high expense for a typical ICP-MS lab has restricted their wide application. Amongst the various absorbents used for FI-ICP-MS, Toyopearl AF Chelate 650M^®^ resin, featuring iminodiacetate functional groups, can sequester all YREEs and offers stability (no shrinkage of the resin under both strong acid and high salt environments), while remaining inexpensive and commercially available [31,37,38,39]. Although Willie and Sturgeon [31] applied this resin for the online determination of YREEs in seawater using FI-ICP-TOF-MS, the relatively high LODs and the poor sample throughput (5 h^−1^) were not attractive for routine measurements.

In this study, based on previous work [40,41], Toyopearl AF Chelate 650M^®^ resin was used to separate YREEs from seawater and a fast FI-ICP-MS method for the online determination of YREEs in seawater was established. The FI system was readily set-up and automated to ensure ease of operation, as well as much cheaper than the FIAS-400 and SeaFAST. Experimental parameters were investigated and optimized to minimize sample consumption and improve the LODs. Finally, the developed method was used for the determination of YREEs in estuarine and coastal seawaters.

## 2. Results and Discussion

### 2.1. Effects of Sample Loading Rate and Time

The sample loading rate and time determine the volume of sample analyzed. To optimize the sample loading rate, 7.5 mL seawater (salinity = 33) was processed through the minicolumn using flow rates ranging from 1.5 to 4.0 mL min^−1^. For all YREEs, it was found that peak areas decreased with increasing loading rate. When the loading rate was 2.0 mL min^−1^, response from Ce dropped the most, to about 89% (Supplementary Information Table S1) of the peak area achieved at 1.5 mL min^−1^ (generally the rate of sample passing a column packed with Toyopearl AF Chelate 650M^®^ resin by gravity is 1.5 mL min^−1^, under which the YREEs can be 100% retained). Taking the analysis time and retention efficiency into consideration, 2.0 mL min^−1^ was subsequently used as the loading rate. 

Theoretically, the peak area response should increase linearly with loading time. In this study, loading times from 120 s to 540 s were examined, with results showing that the relative coefficient (R^2^) between the peak areas and loading times were all >0.9934 for all YREEs over the entire time range (Supplementary Information Table S2), indicating that the capacity of the minicolumn would not be overloaded even by processing 18 mL of high salinity seawater. In order to shorten the running time and improve throughput, 180 s was selected as the sample loading time, i.e., 6 mL sample was consumed. Loading time can be extended if the YREEs concentrations are significantly lower. 

### 2.2. Influence of Interferences and Effect of Rinsing Conditions

Although ICP-MS detect YREEs may suffer from polyatomic interference, proper selection of target isotopes may help minimize such effects. However, interferences from lower REE oxides such as ^143^Nd^16^O^+^ on ^159^Tb, ^147^Sm^16^O^+^ on ^163^Dy, ^149^Sm^16^O^+^ on ^165^Ho, ^150^Nd^16^O^+^ and ^150^Sm^16^O^+^ on ^166^Er, ^153^Eu^16^O^+^ on ^169^Tm, ^159^Tb^16^O^+^ on ^175^Lu are inevitable. Nevertheless, the oxide level was minimized during the tuning step by optimizing the ICP-MS parameters to ensure ^140^Ce^16^O/^140^Ce <2%, plus the concentration variances of YREEs are generally within one order of magnitude, thus corrections for interference caused by light YREEs oxides were not considered, and polyatomic interferences such as ^131^Ru^16^O^+^ on ^147^Sm, ^140^Ce^35^Cl^+^ on ^175^Lu, and ^135^Ba^16^O^+^ on ^151^Eu can be overcome using collision gas (He) mode [26,31,33]. 

**Figure 1:**
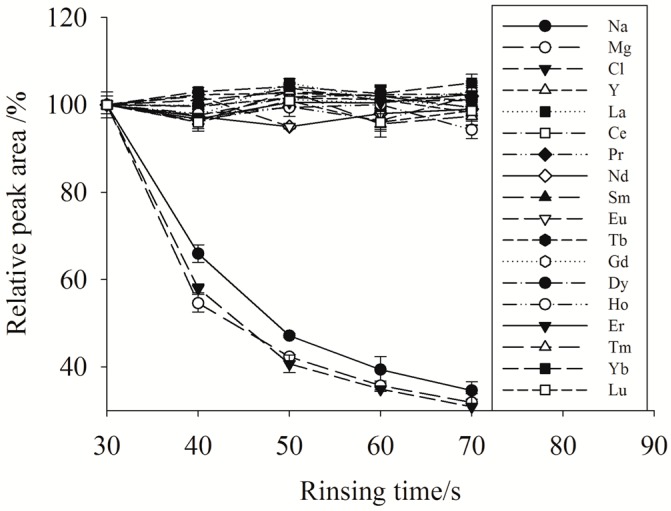
Effect of rinse time on Na, Mg, Cl and YREEs peak areas; test sample salinity = 33. The relative peak areas were normalized to that obtained using a rinsing time of 30 s.

However, physical interference induced by the salt matrix on instrument should also be resolved, since large amounts of salts (e.g., Na^+^, K^+^, Ca^2+^, Cl^−^) may deposit on the torch and the cones of the ICP-MS to cause considerable signal depression and drift in YREEs response. After sample loading, a mixture of buffer solution and ultrapure water was passed through the minicolumn to remove the residual salts. The flow rate was the same as the loading rate (2.0 mL min^−1^) to reduce flow pulses in the minicolumn and the rinse time ranged from 30 to 70 s was studied. To minimize the rinse time, a test sample of salinity 33 was processed and response of Na, Mg, Cl, Ba and the YREEs were monitored. The relative peak areas (%) of each elements using rinsing time 40–70 s to 30 s are shown in Figure 1. With a rinsing time of 60 s, peak areas of Na, Mg and Cl decreased to 39%, 36% and 35%, respectively. While no significant decrease in peak area was observed with a further 10 s rinsing. The peak areas of Ba were nearly the same as the procedural blank, demonstrating that Ba in the seawater was not retained by the minicolumn, such that its interferences were negligible. All YREEs were approximately 100% recovered irrespective of the rinse time range. Consequently, 60 s was determined to be the optimal rinse time. 

### 2.3. Effects of Eluting Condition

**Figure 2:**
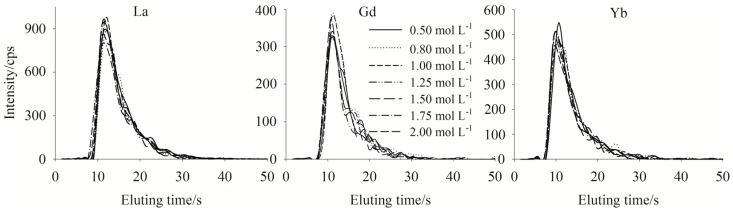
Elution profiles for La, Gd and Yb when eluted with different concentrations of HNO_3_; test sample salinity = 33.

**Figure 3:**
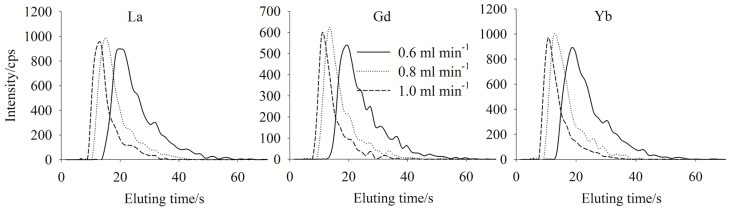
Elution profiles for Y when eluted with 0.8 mol L^−1^ HNO_3_ at different flow rates; test sample salinity = 33.

To ensure the retained YREEs were totally eluted, the concentration of eluent (HNO_3_) and elution rate and time were optimized. Results for La, Gd and Yb are shown in Figure 2 and Figure 3 as examples. The concentrations of HNO_3_ used ranged from 0.5 to 2.0 mol L^−1^. The results showed that there were no significant differences between the peak shapes for the various HNO_3_ concentrations (Figure 2) with the result that 0.8 mol L^−1^ HNO_3_ was selected as the eluent since 0.5 mol L^−1^ HNO_3_ is not strong enough to elute all sequestered trace metals which will stay on the column and compete with YREEs from the next sample to chelate with the resin. While more concentrated HNO_3_ than 0.8 mol L^−1^ was not considered to protect ICP-MS, and our study showed that when used 0.8 mol L^−1^ HNO_3_ as the eluting acid, the retention efficiency of the column would not decrease after 400 runs. As for elution rate, 1.0 mL min^−1^ was selected to obtain the fastest elution (Figure 3), since a higher flow rate may lead to instability of the plasma. With eluting use 0.8 mol L^−1^ HNO_3_ under a flow rate of 1.0 mL min^−1^, 50 s was required to elute all YREEs.

### 2.4. Calibration and Effect of Salinity

**Figure 4:**
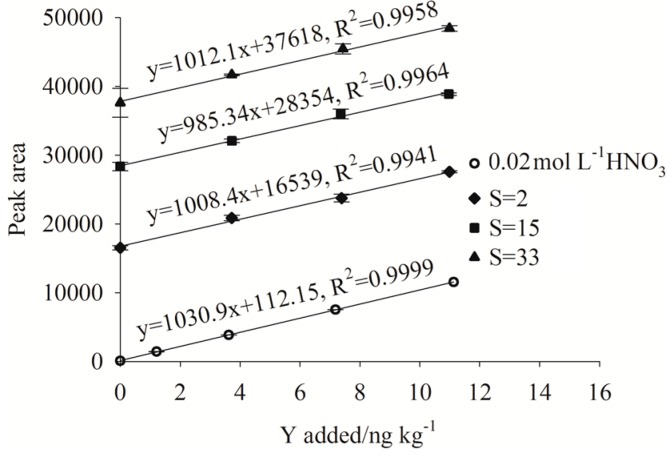
Standard additions calibration curves for Y based on samples having different matrices.

When analyzing high matrix samples, the method of standard addition is often used for quantification to compensate for matrix effects. However, this methodology is tedious and labor intensive. Research by Willie and Sturgeon [31] concluded that the recoveries of YREEs from seawater using Toyopearl AF Chelate 650M^®^ resin were independent of the salinity, and many studies have utilized a simple external calibration based on dilute HNO_3_ for quantification. To confirm this conclusion and expand the simple calibration to seawater samples having a wide range of salinities (i.e., estuarine waters), four calibration curves (0–10 ng kg^−1^) based on four different matrices (0.02 mol L^−1^ HNO_3_, estuarine water sample with salinities of 2, 15 and 33) were prepared and analyzed using FI-ICP-MS. All four samples were using standard additions methodology (briefly, YREEs working standards were added to separate aliquots of a sample, and then the standard-containing samples plus the original sample were analyzed using FI-ICP-MS). The four calibration curves for Y are displayed in Figure 4 as an example.

The slopes of the four curves for each YREEs were tested for significant difference by SPSS (IBM SPSS Statistics 19.0, New York, NY, USA) using covariance analysis and all achieved a σ score (probability value) >0.05 (confidence level), indicating that the four slopes were not statistically different, demonstrating that the retention and elution properties of Toyopearl AF Chelate 650M^®^ were not influenced by salinity. YREEs concentrations were thus calculated using external calibration curves comprising a 0.02 mol L^−1^ HNO_3_ as the matrix.

### 2.5. Analytical Figures of Merit

Using the optimized conditions, about 5.3 min was required for processing a 6 mL sample, resulting in a sample throughput of 11 h^−1^. The linear range of the method was examined using a ten-point external calibration curve with concentrations from 0 to 200 ng kg^−1^. The R^2^ of the 7 ranges (0–5, 0–7, 0–15, 0–25, 0–50, 0–100 and 0–200 ng kg^−1^) were all larger than 0.9917 (except for Tm, the R^2^ of which was 0.9479 for the 0–200 ng kg^−1^ range). Covariance analysis was used to investigate the significant difference between the slopes of the 7 curves. Results showed the σ scores for all YREEs >0.05 (excepted Tm in 0–200 ng kg^−1^ range), indicating no significant differences between the 7 slopes for each YREEs. Therefore, the developed FI-ICP-MS procedure was capable of accurate measurements of YREEs concentration up to 200 ng kg^−1^ (100 ng kg^−1^ for Tm), suitable for the determination of YREEs in almost all seawater samples. 

The precision and accuracy of the method were also examined using samples having differing salinities (salinity = 2, 15 and 33). The repeatability and reproducibility of the method was evaluated via repetitive inter-day (*n* = 5) and separate-day (*n* = 4) measurements of three samples, with inter-day RSDs of 0.3–6% and separate-day RSDs of 2–8%. Spike recovery testing was conducted and recoveries of 93–106% were obtained, confirming the accuracy of the method. Also we tested the performance of the method by analysis some high salinity aged seawater sample (salinity = 35–40, to simulate open ocean water with low level dissolved organic matter), and better RSDs of 0.47–4.81% and recovery of 94.5–104% were obtained, likely due to the aged seawater contained much less dissolved organic matter, which may compete with YREEs to absorb on the resin or compete with the resin to absorb the YREEs, than the estuarine and coastal seawater. These results suggested that the developed method provides satisfactory analytical results for seawater with wide range salinity.

Although Certified Reference Materials for YREEs in seawater (like newly released NASS-7 and some GEOTRACES intercalibration reference materials) were not commercially available at the time of this study, compiled results of multiple reports on YREE concentrations in CASS-4 and SLEW-3 can provide valuable reference data for the validation of the methodology developed in this study [35]. Such reference data, plus that obtained in this study are summarized in Table 1.

The data show good agreement with other studies (except the Y, Sm, Gd, Ho and Tm in CASS-4, whose RSD of this study value and the reference compiled were >5%), indicating the proposed method can provide reliable YREEs concentrations in estuarine and coastal seawater. As to the durability of the minicolumn, no decrease of YREEs retention efficiency was detected after 400 runs. 

The procedural blank was obtained by processing 0.02 mol L^−1^ HNO_3_ (pH ~ 1.6). The LODs of this FI-ICP-MS system were calculated based on 3 s (standard deviation) of 11 procedure blanks. The results are shown in Table 2. The blank and LODs are sufficiently low to permit determination of YREEs in estuarine and coastal seawaters. However if the open ocean seawater was subject to analysis, the HNO_3_ and the buffer should be further purified to reduce the procedure blank. For the HNO_3_, a second or even more times of distillation can be adopted, while for the buffer an additional minicolumn packed with Toyopearl AF Chelate 650M^®^ resin placed on the buffer line can be implemented.

### 2.6. Comparison with Other FI-ICP-MS Systems

Figures of merit of the developed FI-ICP-MS procedures are summarized in Table 3. Compared with other FI systems, this arrangement is easy to construct and short sample duration to ensure high sample throughput (11 h^−1^), and provided excellent LODs based on only 6 mL sample consumption, moreover the total cost of this FI system is only about 20% of the cost of FIAS-400 (let alone the much more expensive seaFAST), making the daily and large scale determination of YREEs in seawater samples relatively inexpensive in a regular ICM-MS lab. Though report of Benkhedda et al. [30] indicated shorter duration (4 min) and less sample consumption than this study, but an ICP-TOF-MS was required and the separation unit based on a knotted reactor was not as easily prepared as a minicolumn used here; results of Wang et al. [34] had the shortest duration (2.8 min) amongst all arrangements, while much more sample was needed and the fast loading rate may risk decreased retention efficiency and pump tubing aging. Advantages of this work were especially obviously when compared with thetudy of Willie and Sturgeon [31], in which the same Toyopearl AF Chelate 650M^®^ absorbent was used, while the sensitivity provided by the ICP-TOF-MS was much lower than the quadrupole ICP-MS used in our work. 

### 2.7. Applications

**Figure 5:**
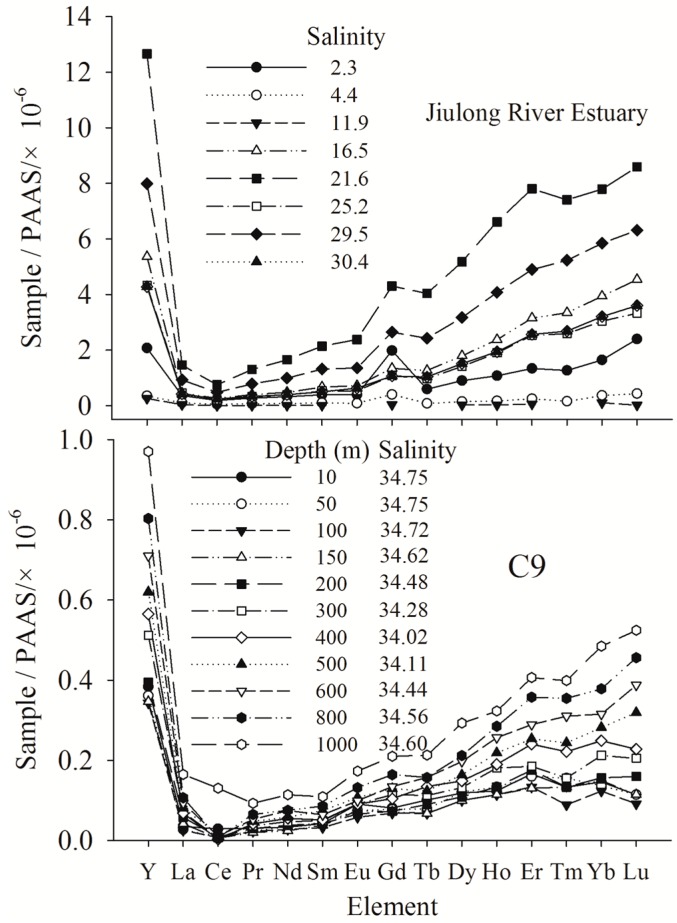
PAAS normalized YREEs patterns in surface water of Jiulong River Estuary and in station C9 (22°07′13″ N, 118°24′41″ E) of Taiwan Strait.

The established method was used for the determination of YREEs in seawater collected from the Jiulong River Estuary and the Taiwan Strait. Results are presented in Supplementary Information Tables S3 and S4. Post-Archean Australian Shales (PAAS) [42] normalized YREEs distributions patterns of the Jiulong River Estuary and the Taiwan Strait are plotted in Figure 5. 

Relatively flat YREEs patterns were observed in samples having salinities of 4.4 and 11.9 from the Jiulong River Estuary, while all YREEs patterns show obviously negative Ce anomaly and positive Gd anomaly (except salinity 11.9). The latter could be attributed to anthropogenic Gd discharge (e.g., MRI contrast reagent). In the seawater of Taiwan Strait, slightly negative Ce anomalies were obtained from the YREEs patterns (except the surface and bottom water), which are commonly observed in the world oceans [6]. The geochemistry of YREEs in the Jiulong River Estuary and the Taiwan Strait will be further studied in future work.

## 3. Materials and Methods 

### 3.1. Reagents and Samples

All solutions were prepared with ultrapure water (18.20 MΩ cm, Millipore, Darmstadt, Germany). Trace metal free nitric acid was obtained by purifying nitric acid (Merck, Darmstadt, Germany) using a sub-boiling distillation system. Standard stock solutions of YREEs (1000 ppm) were obtained from the National Institute of Metrology (Beijing, China). Working standards were prepared via serial dilutions of the stock solution with 0.02 mol L^−1^ purified HNO_3_ (equal to acidified sample pH ~ 1.6). Ammonium acetate (NH_4_Ac) buffer solution was prepared by mixing 30 mL aqueous ammonia (Sinopharm Chemical Reagent Co., Nanjing, China) and 20 mL glacial acetate acid (HAc, Sinopharm Chemical Reagent Co., Nanjing, China) and diluting to 1 L using ultrapure water; the pH was subsequently adjusted to 5.5 ± 0.2 with HAc or NH_4_OH [31]. The buffer solution was further purified to remove potential YREEs by passing it through a column packed with Toyopearl AF Chelate 650M^®^ resin. Two Certified Reference Materials (SLEW-3 and CASS-4) were purchased from the National Research Council Canada (Ottawa, Canada). Estuarine samples (salinity of 2 and 15) collected from the Jiulong River Estuary and coastal seawater (salinity = 33) collected from the South China Sea were used to optimize the method, All samples were acidified to pH ~ 1.6 using purified HNO_3_ after filtered using 0.45 μm polycarbonate membranes. Trace metal clean procedures were used for the water sample collection.

All reagents and samples were stored in fluorinated ethylene propylene, low-density polyethylene or polypropylene acid washed bottles (Nalgene, Rochester, NY, USA). The cleaning procedure for all labware is detailed in Wen et al. [43].

### 3.2. Instrumentation

An Agilent 7700× ICP-MS (Agilent, Tokyo, Japan) operating in time-resolved-analysis mode was used for the measurement of YREEs. The ICP-MS was equipped with an octopole reaction/collision system which was employed to help overcome oxide and polyatomic interferences. The operating conditions were daily optimized with a 1 µg L^−1^ tuning solution (Co, Y, In, Tl, Ce) in the eluting acid at a flow rate equal to the elution rate. The typical operating parameters are summarized in Table 4. 

### 3.3. FI System and FI-ICP-MS Analysis Procedure

**Figure 6:**
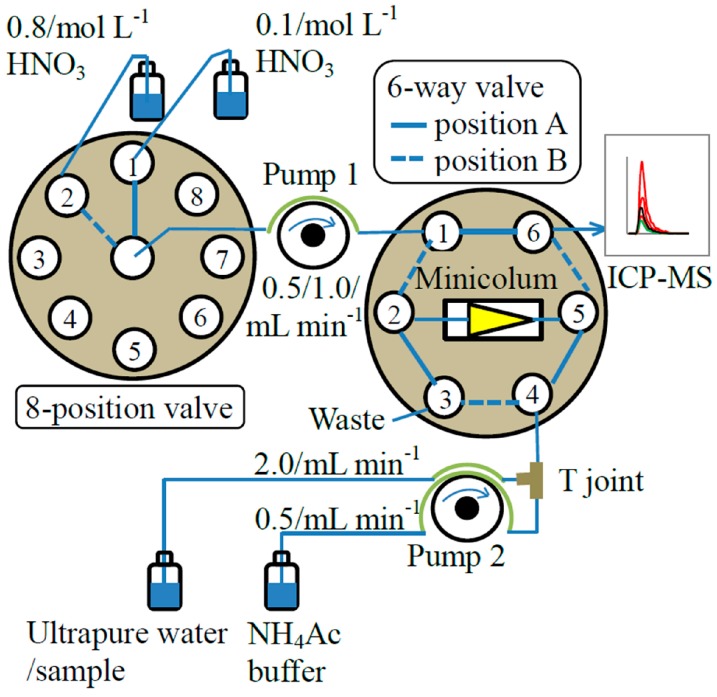
Schematic of the FI system and the FI-ICP-MS procedure.

The construction of the FI system used in this study is shown in Figure 6. Apart from the metal free minicolumn assembly (MC-2CNME, Global FIA, Fox Island, WA, USA) with a tapered inner chamber (2 cm long with 27 µL internal volume) packed with Toyopearl AF Chelate 650M^®^ (particle size: 40–90 µm; Tosoh Bioscience GmbH, Griesheim, Germany) resin and the T joint (i.d. 0.75 mm, VICI, Houston, TX, USA), all other parts of the FI system were the same as those used in our previous study [40,41]. The FI system was controlled using a computer running LabVIEW program (National Instruments, Austin, TX, USA). The schematic of FI-ICP-MS procedure is given in Figure 6 and the optimized FI program is summarized in Table 5.

A run cycle comprises four steps: step 1, conditioning, buffer solution and ultrapure water are mixed at the T joint and then passed through the minicolumn; step 2, loading, sample tube is placed into the sample bottle, sample (pH ~ 1.6) is online buffered to pH 5.5 ± 0.2 before entering the minicolumn and YREEs are retained on the column while the matrix salts pass to waste; step 3, rinsing, sample tube is transferred to the ultrapure water bottle and the mixture of buffer solution and ultrapure water is passed through the minicolumn to remove residual salts; step 4, 8-position valve is switched from position 1 to position 2 and 6-way valve is switched from position A to position B, 0.8 mol L^−1^ HNO_3_ is pumped through the minicolumn in the reverse direction to elute the sequestered YREEs to the ICP-MS, and the data acquisition by the ICP-MS is manually activated at the same time. The elution profiles are recorded and the peak areas are integrated using the Agilent MassHunter workstation (Agilent, Santa Clara, CA, USA). The integration range was determined based on the comparison of YREEs signal intensity between the sample and the baseline (0–5 cps). The concentrations of the YREEs are determined using both standard addition as well as external standard calibration (see details in Section 2.4. Calibration and effect of salinity).

## 4. Conclusions

An automated FI system coupled online with ICP-MS to determine YREEs in seawater was developed. The components of the FI system in this work are all commercially available and the FI system is easy and cheap to assemble. With low LODs (0.002–0.078 ng kg^−1^), the method only needs 6 mL of sample and achieves accurate and fast sample analysis (11 h^−1^), making the regular monitoring of YREEs in seawater affordable. The analytical results of YREEs in CASS-4 and SLEW-3 confirmed that the proposed method can provide reliable results. The proposed method has been successfully applied to the determination of YREEs in seawater from the Jiulong River Estuary and Taiwan Strait, and the procedure blank can be further reduced to meet the requirement of measurement of open ocean seawater by the further purification of HNO_3_ and the buffer. The developed FI system can also be used as a preconcentration manifold for the offline detection (batch method) of not only YREEs but also other transition metals (Fe, Mn, Cu, Zn, etc.), and both the online and offline methods will be used in future work for trace metal detection in seawater.

## Figures and Tables

**Table 1:** YREEs concentrations in CASS-4 (Salinity = 30.7) and SLEW-3 (Salinity = 15) from references and this study.

Elements	CASS-4 (ng kg^−1^)			SLEW-3 (ng kg^−1^)		
	Reference Compiled ^a^	This Study ^b^	RSD ^c^ (%)	Reference Compiled ^d^	This Study ^b^	RSD ^c^ (%)
Y	20.93 ± 0.40	18.89 ± 0.12	7.25	40.55 ± 2.05	38.10 ± 2.39	4.41
La	9.37 ± 0.38	9.96 ± 0.15	4.33	7.80 ± 0.13	8.22 ± 0.25	3.71
Ce	4.69 ± 0.92	4.90 ± 0.07	3.13	7.08 ± 0.68	7.19 ± 0.45	1.06
Pr	1.33 ± 0.06	1.37 ± 0.01	2.10	1.68 ± 0.05	1.64 ± 0.03	1.78
Nd	5.39 ± 0.47	5.49 ± 0.04	1.30	8.18 ± 0.35	7.97 ± 0.19	1.82
Sm	5.55 ± 0.17	6.00 ± 0.21	5.45	7.10 ± 0.15	7.38 ± 0.21	2.69
Eu	0.23 ± 0.03	0.23 ± 0.02	0.00	0.54 ± 0.08	0.55 ± 0.02	1.72
Gd	1.29 ± 0.1	1.46 ± 0.04	8.74	3.09 ± 0.01	3.20 ± 0.06	2.36
Tb	0.20 ± 0.03	0.20 ± 0.01	0.00	0.45 ± 0	0.43 ± 0.02	2.94
Dy	1.41 ± 0.08	1.42 ± 0.05	0.38	3.37 ± 0.02	3.33 ± 0.08	0.95
Ho	0.38 ± 0.05	0.35 ± 0.02	6.32	0.91 ± 0	0.91 ± 0.07	0.13
Er	1.20 ± 0.1	1.27 ± 0.08	4.15	2.71 ± 0.01	2.78 ± 0.05	1.72
Tm	0.23 ± 0.07	0.20 ± 0.02	10.75	0.37 ± 0	0.35 ± 0.01	3.93
Yb	1.21 ± 0.14	1.16 ± 0.01	3.29	1.95 ± 0.14	1.85 ± 0.06	3.85
Lu	0.20 ± 0.03	0.19 ± 0.01	4.56	0.31 ± 0.03	0.30 ± 0.01	3.51

^a^ Mean ± 1 standard deviation, *n* = 6, based on results in references [16,19,20,21,25,27]; ^b^ Mean ± 1 standard deviation, *n* = 5; ^c^ Relative standard deviation of this study from the data of reference compiled; ^d^ Mean ± 1 standard deviation, *n* = 3, based on results in references [16,19].

**Table 2:** Procedural blanks and LODs.

Elements	Blank ^a^ (ng kg^−1^)	LODs (ng kg^−1^)
Y	0.126 ± 0.023	0.034
La	0.172 ± 0.05	0.045
Ce	0.61 ± 0.112	0.078
Pr	0.038 ± 0.023	0.019
Nd	0.124 ± 0.038	0.048
Sm	0.046 ± 0.021	0.027
Eu	0.007 ± 0.004	0.009
Gd	0.05 ± 0.01	0.022
Tb	0.007 ± 0.003	0.003
Dy	0.03 ± 0.007	0.021
Ho	0.006 ± 0.003	0.003
Er	0.02 ± 0.007	0.012
Tm	0.003 ± 0.003	0.002
Yb	0.009 ± 0.007	0.005
Lu	0.002 ± 0.002	0.002

^a^ Mean ± 1 standard deviation, *n* = 5.

**Table 3:** Comparison with other FI-ICP-MS methodologies for the determination of YREEs in seawater.

Loading	Eluting			Duration (min)	Absorbent	Sample (mL)	LODs (ng kg^−1^)	Reference
Rate (mL min^−1^)	Rate (mL min^−1^)	Time (s)	HNO_3_ (mol L^−1^)					
5	1.2	80	1.0	~5.5	- ^a^	10	0.06–0.27	[8]
12	1.5	300	2.0	~12	Amberlite XAD-7 + 8HQ	100	0.002–0.016 ^b^	[9]
2.0	1.0	30	0.1	>30	APAR ^c^	60	0.001–0.013	[12]
2	1.0	100	0.8 ^d^	~9.5	I-8-HQ ^e^	3	0.06–0.6	[29]
4.4	0.8	90	0.4	~4	PMBP ^f^	2.2	0.003–0.04	[30]
5	1.5	30	1.5	12	Toyopearl AF Chelate 650M^®^	50	0.02–0.29	[31]
3.2	1.7	61	1.0	7	Muromac A-1	6.4	0.04–0.251	[32]
3.2	2.0	60	1.4	7	MAF-8HQ ^g^	6.4	0.11–0.30	[33]
7.4	0.5	35	0.9	2.8	M-PTFE ^h^	14.8	0.001–0.02	[34]
5	0.5	120	2	6	Nobias chelate PB1M	10	0.005–0.09	[35]
1.0	0.3	5	1.5 ^i^	15	-	7	0.001–0.036	[36]
2.0	1.0	60	0.8	~5.3	Toyopearl AF Chelate 650M^®^	6	0.002–0.078	This study

^a^ precipitation reagent; ^b^ only Eu, Tb, Ho, Tm, Lu detected; ^c^ alkyl phosphinic acid resin; ^d^ 2 mol L^−1^ HCl + 0.8 mol L^−1^ HNO_3_; ^e^ 8-hydroxyquinoline; ^f^ 1-phenyl-3-methyl-4-benzoylpyrazol-5-one; ^g^ 8-quinoline-immoblized fluorinated metal alkoxide glass; ^h^ polytetrafluoroethylene; ^i^ 1.5 mol L^−1^ HNO_3_ + 0.4% HAc.

**Table 4:** Typical ICP-MS operating conditions.

Rf Power	1500 W
Plasma gas	15.0 L min^−1^
Auxiliary gas	1.0 L min^−1^
Carrier gas	0.85 L min^−1^
Collision gas (He)	4.1 mL min^−1^
Integration time	0.1 s per isotope
Sampling depth	8 mm
Target isotopes	^89^Y ^139^La ^140^Ce ^141^Pr ^143^Nd ^147^Sm ^151^Eu ^157^Gd ^159^Tb ^163^Dy ^165^Ho ^166^Er ^169^Tm ^174^Yb ^175^Lu

**Table 5:** Typical flow injection program and valve position description.

Step	Duration/s	Pump 1/mL min^−1^	Pump 2/mL min^−1^	8-Position Valve	6-Way Valve
Conditioning	20	0.5	1.5	1	A
Loading	180	0.5	2.0	1	A
Rinsing	60	0.5	2.0	1	A
Eluting	50	1.0	0	2	B
Return to conditioning					

## Supplementary Materials

The following are available online, Table S1: Percentage of REEs peak area (%) of different loading rates to peak area of 1.5 mL min^-1^. Table S2: Relative coefficients (R^2^) between REEs peak areas and loading times. Table S3: REEs concentrations measured by the presented method from samples collected in the Jiulong River Estuary (water samples were collected in April 2015). Table S4: REEs concentrations measured by the presented method from samples collected in Taiwan Strait (seawaters were collected at the station C9, 22°07′13″ N, 118°24′41″ E, in April 2014).
