# Supplementary material for: Fast Determination of Yttrium and Rare Earth Elements in Seawater by Inductively Coupled Plasma-Mass Spectrometry after Online Flow Injection Pretreatment
Source: Molecules. 2018 Feb 23;23(2):489. doi: 10.3390/molecules23020489 (PMC6017308; doi:10.3390/molecules23020489)
Supplement: Supplementary file 1 [file molecules-23-00489-s001.pdf]

# Supplementary information

**Table S1 Percentage of REEs peak area (%) of different loading rates to peak area of 1.5 mL min$^{-1}$.**

| Rate/ mL min$^{-1}$ | Y | La | Ce | Pr | Nd | Sm | Eu | Gd | Tb | Dy | Ho | Er | Tm | Yb | Lu |
|---|---|---|---|---|---|---|---|---|---|---|---|---|---|---|---|
| 2 | 95.96 | 92.62 | 89.46 | 94.56 | 92.86 | 96.85 | 102.03 | 96.35 | 99.09 | 98.76 | 99.66 | 96.97 | 100.45 | 98.53 | 92.49 |
| 2.5 | 94.21 | 82.54 | 82.69 | 88.17 | 87.12 | 96.34 | 100.10 | 95.68 | 95.42 | 96.71 | 95.96 | 94.38 | 98.17 | 97.10 | 96.28 |
| 3 | 89.44 | 77.19 | 78.37 | 82.05 | 83.43 | 94.48 | 97.45 | 93.85 | 93.26 | 90.22 | 94.91 | 90.66 | 91.93 | 94.79 | 91.30 |
| 3.5 | 89.44 | 77.19 | 78.37 | 82.05 | 83.43 | 94.48 | 97.45 | 93.85 | 93.26 | 90.22 | 94.91 | 90.66 | 91.93 | 94.79 | 91.30 |
| 4 | 87.53 | 68.23 | 71.99 | 77.03 | 78.65 | 90.92 | 87.62 | 89.66 | 85.86 | 88.98 | 90.31 | 88.33 | 87.82 | 91.16 | 88.57 |

**Table S2 Relative coefficients ($R^2$) between REEs peak areas and loading times.**

| Time/ min | Y | La | Ce | Pr | Nd | Sm | Eu | Gd | Tb | Dy | Ho | Er | Tm | Yb | Lu |
|---|---|---|---|---|---|---|---|---|---|---|---|---|---|---|---|
| 4 | 0.9997 | 0.996 | 0.9997 | 0.9985 | 0.9965 | 0.997 | 0.9943 | 0.9985 | 0.9997 | 1 | 0.995 | 0.9991 | 0.9952 | 0.9991 | 0.9957 |
| 5 | 0.9999 | 0.9984 | 0.9997 | 0.9976 | 0.9934 | 0.9988 | 0.9977 | 0.999 | 0.9994 | 0.996 | 0.998 | 0.9995 | 0.9957 | 0.999 | 0.9972 |
| 6 | 0.9999 | 0.999 | 0.9999 | 0.9987 | 0.9958 | 0.9992 | 0.9952 | 0.9982 | 0.9996 | 0.9974 | 0.9989 | 0.9994 | 0.9978 | 0.9992 | 0.9986 |
| 7 | 1 | 0.9958 | 0.9998 | 0.9991 | 0.9973 | 0.9988 | 0.9965 | 0.999 | 0.9997 | 0.9979 | 0.9994 | 0.9996 | 0.9987 | 0.9992 | 0.9982 |
| 8 | 0.9998 | 0.997 | 0.9982 | 0.9993 | 0.9959 | 0.9984 | 0.9949 | 0.9993 | 0.9996 | 0.9987 | 0.9995 | 0.9995 | 0.999 | 0.9995 | 0.9981 |
| 9 | 0.9999 | 0.9963 | 0.9986 | 0.9995 | 0.9969 | 0.9984 | 0.9958 | 0.9995 | 0.9997 | 0.999 | 0.9989 | 0.9994 | 0.9991 | 0.9996 | 0.9981 |

**Table S3 REEs concentrations measured by the presented method from samples collected in the Jiulong River Estuary (water samples were collected in April 2015)**

| Elements | Sample salinity | | | | | | | |
|---|---|---|---|---|---|---|---|---|
| | 2.3 | 4.4 | 11.9 | 16.5 | 21.6 | 25.2 | 29.5 | 30.4 |
| | REEs concentration/ng $kg^{-1}$ | | | | | | | |
| Y | 56.61 | 9.54 | 7.14 | 146.28 | 345.67 | 118.16 | 218.36 | 116.84 |
| La | 16.14 | 4.56 | 1.67 | 20 | 65.1 | 20.04 | 41.27 | 18.57 |
| Ce | 16.67 | 4.52 | 0.03 | 23.39 | 66.47 | 18.10 | 42.48 | 20.91 |
| Pr | 2.76 | 0.65 | 0.1 | 4.02 | 13.24 | 3.29 | 7.97 | 3.35 |
| Nd | 12.09 | 3.02 | 0.46 | 17.89 | 61.59 | 14.54 | 37.16 | 14.76 |
| Sm | 2.66 | 0.67 | 0.07 | 4.61 | 14.76 | 3.63 | 9.07 | 3.43 |
| Eu | 0.47 | 0.1 | UD[a] | 0.87 | 2.89 | 0.65 | 1.65 | 0.76 |
| Gd | 11.98 | 2.4 | 0.23 | 8.12 | 26.02 | 6.49 | 16.01 | 6.43 |
| Tb | 0.53 | 0.07 | UD | 1.13 | 3.6 | 0.87 | 2.16 | 0.92 |
| Dy | 4.79 | 0.78 | 0.14 | 9.56 | 27.56 | 7.53 | 16.9 | 8.07 |
| Ho | 1.13 | 0.18 | 0.03 | 2.5 | 6.96 | 2 | 4.29 | 2.04 |
| Er | 4.11 | 0.76 | 0.19 | 9.7 | 24.01 | 7.78 | 15.05 | 7.87 |
| Tm | 0.57 | 0.07 | UD | 1.51 | 3.34 | 1.17 | 2.36 | 1.21 |
| Yb | 4.94 | 1.1 | 0.31 | 11.87 | 23.47 | 9.17 | 17.61 | 9.65 |
| Lu | 1.05 | 0.19 | 0.01 | 1.99 | 3.77 | 1.46 | 2.77 | 1.58 |

[a] UD=under detection limit.

**Table S4 REEs concentrations measured by the presented method from samples collected in Taiwan Strait (seawaters were collected at the station C9, 22°07’13”N, 118°24’41”E, in April 2014.)**

| Elements | Sample depth/m | | | | | | | | | | |
|---|---|---|---|---|---|---|---|---|---|---|---|
| | 10 | 50 | 100 | 150 | 200 | 300 | 400 | 500 | 600 | 800 | 1000 |
| | REEs concentration/ng $kg^{-1}$ | | | | | | | | | | |
| Y | 10.48 | 9.87 | 9.38 | 9.46 | 10.78 | 13.98 | 15.43 | 16.93 | 19.39 | 21.94 | 26.49 |
| La | 1.57 | 1.21 | 1.12 | 1.85 | 2.56 | 3.12 | 3.03 | 3.59 | 4.14 | 4.74 | 7.33 |
| Ce | 2.54 | 0.66 | 0.63 | 0.51 | 0.74 | 0.58 | 0.29 | 1.27 | 0.94 | 0.59 | 11.49 |
| Pr | 0.31 | 0.24 | 0.23 | 0.2 | 0.3 | 0.37 | 0.42 | 0.48 | 0.53 | 0.66 | 0.94 |
| Nd | 1.24 | 1.26 | 1.03 | 0.91 | 1.41 | 1.74 | 2.05 | 2.16 | 2.78 | 2.8 | 4.24 |
| Sm | 0.29 | 0.31 | 0.22 | 0.26 | 0.29 | 0.34 | 0.35 | 0.51 | 0.44 | 0.59 | 0.75 |
| Eu | 0.11 | 0.09 | 0.07 | 0.09 | 0.08 | 0.11 | 0.11 | 0.13 | 0.12 | 0.16 | 0.21 |
| Gd | 0.49 | 0.47 | 0.41 | 0.43 | 0.44 | 0.69 | 0.63 | 0.78 | 0.81 | 0.99 | 1.27 |
| Tb | 0.09 | 0.07 | 0.06 | 0.06 | 0.08 | 0.1 | 0.12 | 0.11 | 0.14 | 0.14 | 0.19 |
| Dy | 0.63 | 0.64 | 0.53 | 0.53 | 0.58 | 0.7 | 0.79 | 0.87 | 1.05 | 1.13 | 1.56 |
| Ho | 0.13 | 0.14 | 0.12 | 0.12 | 0.14 | 0.19 | 0.2 | 0.23 | 0.27 | 0.3 | 0.34 |
| Er | 0.51 | 0.49 | 0.41 | 0.4 | 0.54 | 0.57 | 0.74 | 0.78 | 0.89 | 1.1 | 1.25 |
| Tm | 0.06 | 0.07 | 0.04 | 0.06 | 0.06 | 0.07 | 0.1 | 0.11 | 0.14 | 0.16 | 0.18 |
| Yb | 0.44 | 0.41 | 0.37 | 0.45 | 0.47 | 0.64 | 0.75 | 0.85 | 0.95 | 1.14 | 1.46 |
| Lu | 0.05 | 0.05 | 0.04 | 0.05 | 0.07 | 0.09 | 0.1 | 0.14 | 0.17 | 0.2 | 0.23 |
